# miR-429 inhibits migration and invasion of breast cancer cells *in vitro*

**DOI:** 10.3892/ijo.2014.2759

**Published:** 2014-11-17

**Authors:** ZHI-BIN YE, GANG MA, YA-HUI ZHAO, YUN XIAO, YUN ZHAN, CHAO JING, KAI GAO, ZHI-HUA LIU, SHENG-JI YU

**Affiliations:** 1 Department of Orthopedics, Cancer Hospital (Institute), Chinese Academy of Medical Sciences and Peking Union Medical College, Beijing 100021, P.R. China; 2 The State Key Laboratory of Molecular Oncology, Cancer Hospital (Institute), Chinese Academy of Medical Sciences and Peking Union Medical College, Beijing 100021, P.R. China; 3 College of Bioinformatics Science and Technology, Harbin Medical University, Harbin, Heilongjiang 150081, P.R. China; 4 Institute of Laboratory Animal Sciences, Chinese Academy of Medical Sciences and Peking Union Medical College, Beijing 100021, P.R. China

**Keywords:** miR-429, ZEB1, CRKL, breast cancer bone metastasis

## Abstract

Accumulating evidence indicates that microRNAs (miRNAs) are involved in regulating cancer invasion and metastasis, and an increasing number of research demonstrates that miRNAs can promote or inhibit cell motility depending on genetic background of different cancers and the microenvironment. In the present study, we established an *in vivo* bone metastasis model of breast cancer by injecting MDA-MB-231 cells into the left ventricle of nude mice, and then screened the differentially expressed miRNAs between parental and bone-metastatic MDA-MB-231 cells using miRNA array. The results revealed that decreased expression of miR-429 was probably involved in negatively regulating bone metastasis of breast cancer cells. On the other hand, overexpression of miR-429 in MDA-MB-231 cells remarkably suppressed invasion *in vitro*. We identified ZEB1 and CRKL as potential targets of miR-429 by analyzing combined results from *in silico* search and global expression array of the same RNA samples. Immunoblot assay confirmed that miR-429 reduced their expression at protein level. Taken together, our results offer an opportunity for further understanding of the recondite mechanisms underlying the bone metastasis of breast cancer.

## Introduction

Skeleton is the most common site of metastasis in breast cancer, which occurs in over 70% of patients suffering this disease. Bone metastasis, which seriously deteriorates the patients’ life quality, is life-threatening and leads to several complications or skeletal-related events, such as pathologic fracture, pain, disability and nerve compression (1). Although huge efforts have been made in breast cancer research, what we can do is still limited with respect to bone metastasis of breast cancer. Safe and effective treatment to prevent or treat bone metastasis require sufficient validated targets. Hence, further insights into the molecular mechanisms underlying the bone metastasis of breast cancer are urgently needed (2).

The spreading from primary site to distant bones of breast cancer cells is very complex involving invasion, intravasation and extravasation of capillary vascular system, and locating at as well as flourishing in bones. Especially, various factors that promote cancer cell migration and invasion play a vital role in the process. It is widely accepted that tumor cells could travel to distant organs because of some acquired genetic alterations that facilitate invasion and metastasis (3).

Recent investigations in the field of cancer cell metastasis rely heavily on the availability of *in vivo* animal models. An ideal animal model should mimic the process of breast cancer cells developing spontaneously metastasis in distant organs. However, the animal models of bone metastasis we can build just represent unique stages of human breast cancer bone metastasis, for this phenomenon occurs rarely in animals (4,5).

miRNA is a class of endogenous small non-coding RNAs which could modulate gene expression. In almost all cases, these RNAs negatively regulate gene expression via RNA-RNA binding to the 3′ untranslated region of target mRNAs in a manner of imperfect complementary, which then leads to either mRNA degradation or translational inhibition. Accumulating evidence demonstrates that miRNAs play crucial roles in tumor initiation and progression, which is exemplified by the ability to regulate every aspect of tumor pathological process including proliferation, migration, invasion, differentiation and escape from senescence and apoptosis (6–9). For example, aberrant expression of miR-106b promotes hepatocellular carcinoma cell migration *in vitro* and metastasis *in vivo* by stimulating epithelial-mesenchymal transition (10). miR-145, on the other hand, inhibits the migration and invasion of metastatic melanoma cells (11).

Several miRNAs have been found to be involved into breast cancer metastasis. For instance, miR-720 suppresses invasion and migration in breast cancer cells by targeting TWIST1 (12). miR-29b regulates migration of human breast cancer cells by modulating PTEN expression (13). In the present study, we established a bone metastasis mouse model by injecting MDA-MB-231 cells into the left ventricle and then compared the differentially expressed miRNAs between the parental malignant cells and the metastatic cells derived from the bones. The distinct expression pattern of the miRNAs provides important insight into the molecular mechanism of bone metastasis of breast cancer. Especially, we focus on the biological function of miR-429 through its target genes ZEB1 and CRKL.

## Materials and methods

### Cell culture and animals

Human mammary carcinoma cell line MDA-MB-231 was purchased from the American Type Culture Collection (ATCC, Manassas, VA, USA) and the cells cultured in the Leibovitz L-15 medium supplemented with 10% fetal bovine serum (FBS), streptomycin (100 mg/ml) and penicillin (100 U/ml). The same culture medium, adding 200 μg/ml G418 (Life Technologies, Carlsbad, CA, USA) was also applied to parental MDA-MB-231 (231-P) cells and the metastatic subline of MDA-MB-231 (231-B) cells derived from the bones of our animal models, both of which had stable luciferase expression. All these cells were maintained in the incubator without gas exchange with outside air.

Five-week-old female Balb/c nude mice were purchased from Vital River Laboratories (Beijing, China) and bred in the SPF Animal Center of Cancer Institute and Hospital, Chinese Academy of Medical Sciences. All the animal experiments were approved by the Institutional Review Board of the Chinese Academy of Medical Sciences Cancer Institute.

### Generation of lentivirus expression vehicles

To facilitate *in vivo* observation and the following the primary culture of 231-B cells, we constructed a pLVX-IRES-Neo vector containing luciferase gene and then produced the lentivirus by transfecting 6.5 μg pLVX-luciferase-Neo vector and corresponding amounts of package vectors into 293T cells, which were seeded in 10-cm flasks before the day of the transfection, following the standard method of Lipofectamine 2000 (Invitrogen). Forty-eight hours after the transfection, supernatant of these 293T cells were harvested, centrifuged (3,000 rpm, 4°C), filtered using 0.45-μm filter flasks, aliquoted and stored at −80°C (14).

For transduction, 10 μl supernatant containing lentivirus was added into the MDA-MB-231 cells, which were maintained in 2 ml of L-15 complete culture medium. In addition, 8 μg/ml of polybrene (Sigma-Aldrich) was also present in order to aid transduction. Twenty-four hours later, the medium were replaced by fresh L15 complemented with 10% FBS. Simultaneously, 400 μg/ml G418 was used to screen out the uninfected cells.

### Establishment of bone metastasis model

The bone metastasis model of breast cancer was described by Yin and colleagues (15). In general, amounts of 231-P cells were harvested using 0.25% trypsin and 0.53 mM EDTA, resuspended in sterilized PBS, and adjusted to a concentration of 5×10^6^/ml. Then, mice were anesthetized by intraperitoneal injection of 1% (w/v) pentobarbital sodium (90 mg/kg). After confirming that mice were under proper anesthesia, we injected 100 μl of the suspended cells into the left ventricle via the third intercostal space with a 29-gauge needle. A successful injection was confirmed by the pumping of arterial blood into the syringe (16). Mice were sterilized with 70% alcohol, and then were bred in the following weeks.

### In vivo detection of bone metastasis

Mice anesthetized by 1% (w/v) pentobarbital sodium (90 mg/kg) were intraperitoneally injected with 150 mg/kg of D-Luciferin (Perkin-Elmer) in DPBS. Bioluminescence images were acquired between 10 and 15 min after injection using Xenogen Corporation Optical *in vivo* imaging (IVIS Lumina). Image acquisition time at the beginning was 60 sec and then it was reduced in accordance with signal strength to avoid saturation. All images were analyzed with Living Image software. Intensity of bioluminescence was calculated as photons/sec/cm^2^/steradian of a region of interest (ROI). Average background reads were obtained from sites of the same mice without bioluminescence.

Moreover, radiographic analysis was adopted to further confirm the bone metastases of 231-P cells. Mice were anesthetized using 1% (w/v) pentobarbital sodium (90 mg/kg) and arranged in proper position and exposed to μCT at 60 kV for 800 msec using an Inveon MM Gantry-LG CT.

### Primary cell culture of metastatic breast tumor cells

To isolate tumor cells from the osteolytic lesions, mice with overt bone metastases were sacrificed, and then the affected hindlimbs were separated from the body at the joints. Both ends of the long bones were cut open after skin and muscle was removed using a scalpel. Mouse bone marrow cells as well as tumor cells were washed out by PBS using a 1-ml syringe with a 29G needle. All those cells were collected by centrifuging and washed with PBS before being cultured in 5-cm plates using L-15 medium supplemented with 10% FBS.

Mouse bone marrow cells that failed to attach to plates and thus could be washed off with PBS after the other cells (almost all of them are tumor cells) adhered to culture plates. After continuous culture using 400 μg/ml G418 for more than 1 week, we obtained the subline of bone metastasis named as 231-B cells, which were verified by measuring the luciferase activity (Promega).

### RNA extraction and expression profiles of miRNA and mRNA

Cells with about 80% confluence on 6-well plates were washed twice using ice-cold PBS, and 1 ml TRIzol (Invitrogen) was added to a well to obtain RNA. Cell lysate in TRIzol was stored at −80°C and then sent for screening differentially expressed miRNA and mRNA using Affymetrix GeneChip miRNA 3.0 Arrays and Human mRNA 4×180K (Agilent Technologies). The quantity and quality of extracted RNA were analyzed by CapitalBio. All data were normalized and further analyses were carried out by the College of Bioinformatics Science and Technology, Harbin Medical University.

### Reverse transcription of RNA and real-time PCR

Total RNA was obtained following the standard method of TRIzol extraction. cDNA was synthesized using Quant cDNA with random primers (Tiangen). Moreover, miRNAs were reverse-transcribed following the instructions with minor alteration (17). In general, one specific reverse primer targeting individual miRNA was designed to complete the reverse transcription. Then, one miRNA-specific forward primer and one universal reverse primer were used in the subsequent real-time PCR detection. Real-time PCR was completed with SYBR Premix Ex Taq™ II (Takara). The PCR procedure followed the instructions of Takara in StepOne Plus Real-time PCR system (Applied Biosystems). The results were analyzed using the 2^−ΔΔCt^ method (18).

Primers used in this study were the following: miR-429 forward, 5′-ACACTCCAGCTGGGTAATACTGTCTGGTAA-3′ and miR-429 reverse, 5′-CTCAACTGGTGTCGTGGAGTCGGCAATTCAGTTGAGACGGTTTT-3′, universal reverse, 5′-TGGTGTCGTGGAGTCG-3′; U6 forward, CTCGCTTCGGCAGCACA and U6 reverse, AACGCTTCACGAATTTGCGT.

### Transfection of microRNA mimics

HiPerFect agent (Qiagen) was used to deliver miRNA mimics into cells maintained in 6-well plates. According to the manufacturer’s instruction, cells were grown to ~70% confluence and 20 nM miRNA mimics and 12 μl of transfection agent was mixed thoroughly in 100 μl of Opti-MEM. After 10 min at room temperature, the mixture was added into cells. The subsequent experiments were performed 48 h after the transfection.

### Cell invasion assays

The upper chambers of Transwells with 8-μm membrane pores (Corning) were pre-coated with 60 μl Matrigel matrix gel (BD Biosciences) at least 1 h before seeding of the tested cells. A total of 3×10^4^ 231-B cells in 100 μl of L15 medium without FBS were added into the upper chambers and 600 μl of L15 medium with 10% FBS was placed to lower chambers as chemoattractant. Twelve hours later, the upper chambers were removed from lower chambers and then wiped using cotton swabs. The invaded cells were fixed using methanol at room temperature for 15 min, visualized and quantified using crystal violet. Three fields of each chamber were photographed (x10 magnification) and the results were from duplicate chambers and are presented as mean ± SD. This experiment was independently repeated at least twice.

### Immunoblot assay

Cells reaching 80% confluence on 6-well plates were washed twice using ice-cold PBS and 80–100 μl cell lysis buffer (50 mM Tris-HCl, 150 mM NaCl, 1 mM EDTA, 1% Triton-100) with protease inhibitor cocktail (Complete Mini EDTA-free; Roche) added into plates. On ice, cells were carefully collected with scrapers and were subjected to lysis for 30 min. Proper amount of Laemmli buffer was added into 20–30 μg cell lysate and boiled at 95°C for 5 min. Then the denaturalized protein was resolved on a 10% gel. After being transferred to PVDF membrane, the protein blot was detected using corresponding antibodies. The immunoblot images were acquired and analyzed using ImageQuant LAS 4000 System. The primary antibodies used in this experiment were ZEB1 and β-actin.

### Statistical analyses

GraphPad 6 was used to evaluate the statistical significance of data. Unless otherwise indicated, t-test was adopted to calculate the statistical significance. A P-value <0.05 was considered significant.

## Results

### Breast cancer cells invade bones of Balb/c nude mice

**Figure 1:**
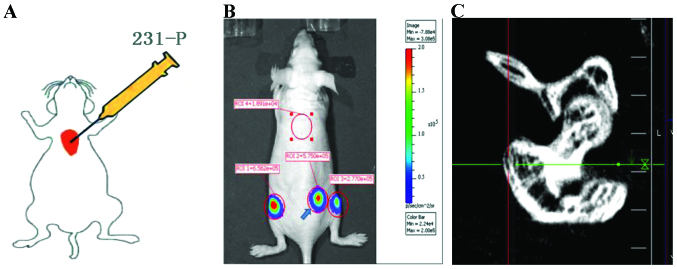
Establishment of the bone metastasis model. (A) Injecting 5×10^5^ 231-P cells into the left ventricle. (B) Twelve weeks later, 3 bone metastasis were detected including distal end of left femur, distal end of right femur and the proximal end of the right femur. The intensity of bioluminescence in bone metastasis was much higher than that of negative background (>10 times). (C) The osteolytic destruction of the proximal end of the right femur which corresponds to bioluminescence signals (arrow in B).

In the present study, we have established a mouse model of bone metastasis. The human mammary carcinoma MDA-MB-231 cells, which constitutively expressed luciferase, were inoculated into the left cardiac ventricle of immunodeficient mice (Fig. 1A). Eight to twelve weeks later, 5 mice eventually developed bone metastasis among the 15 mice under experiment. The bone lesions mainly occur on the long bones of the hind limbs and the ribs, which were observed by Xenogen Corporation Optical *in vivo* imaging and μCT (Fig. 1B and C).

### Primary culture of bone metastatic breast cancer cells

**Figure 2:**
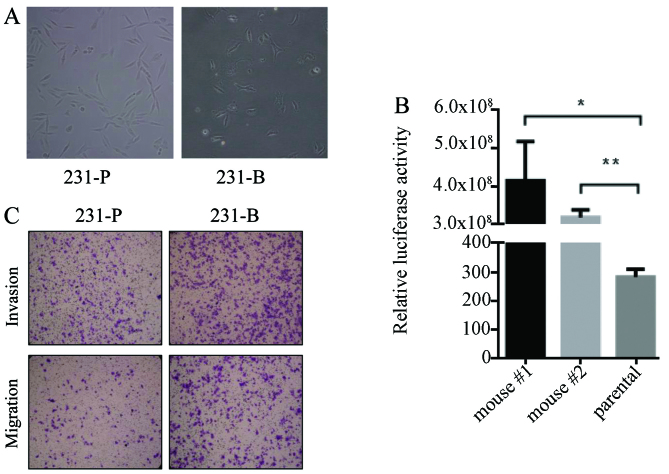
Verification of bone metastatic MDA-MB-231 cells. (A) The distinct morphological characteristics of 231-P cells (left) and 231-B cells (right). (B) MDA-MB-231 cells from bone metastatic lesions of two Balb/c nude mice manifest much stronger luciferase activity than that of cells from the negative control group. (C) *In vitro* Transwell assays show significantly increased migration and invasion of 231-B cells than that of 231-P cells.

After verifying the presence of bone metastases, we collected the metastatic tumor cells from the affected bones. G418 were added into the culture medium to wipe out non-malignant cells. After about 10 days, we obtained 231-B cells (Fig. 2A) of which morphology was different from 231-P cells. Then, we tested the luciferase activity of 231-B cells, obtaining much higher luciferase activity compared with the negative control cells (Fig. 2B). Moreover, *in vitro* invasion assay showed that 231-B cells had stronger invading capability than 231-P cells (Fig. 2C). Thus, we obtained bone metastatic breast tumor cells from the established model.

### Differentially expressed miRNAs of 231-P and 231-B cells

**Figure 3:**
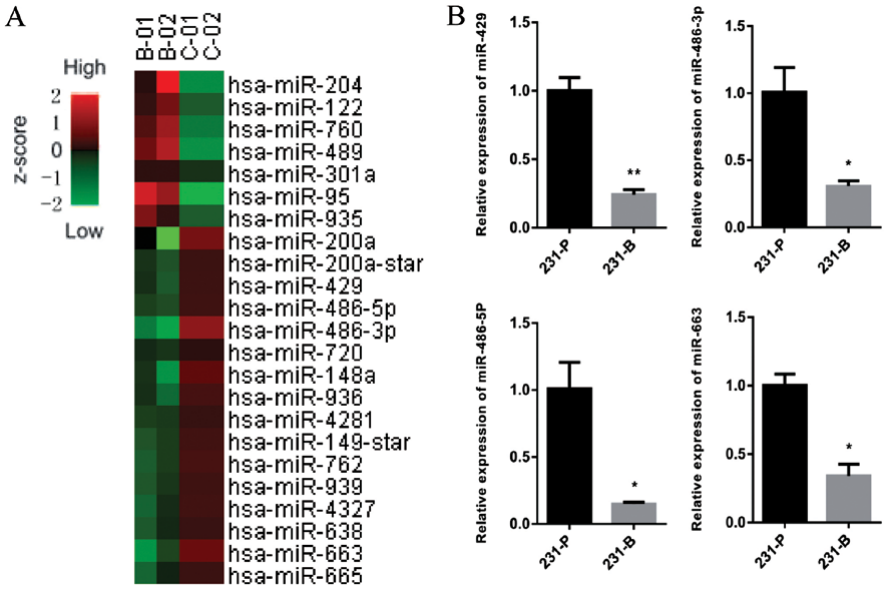
(A) microRNA array shows some of the differently expressed miRNAs. (B) Verifications of microRNA array results by real-time PCR. miR-429, miR-663, miR-486-5p and miR-486-3p showed lower expression in 231-B cells than that in 231-P cells.

RNA samples from 231-P and 231-B cells were prepared and analyzed using Affymetrix GeneChip miRNA 3.0 Arrays. Among the detected miRNAs that amounted to 3,949, 118 miRNAs manifested differential expression between 231-P and 231-B cells, of which 16 upregulated whereas 102 downregulated >2-fold (P<0.05). Some of the differentially expressed miRNA are shown in Fig. 3A. Subsequent GO enrichment analysis showed that the functions of these miRNAs focused on ‘regulation of cell death’ (P=1.49xe^−6^), ‘signaling’ (P=3.25xe^−6^), ‘regulation of localization’ (P=6.31 xe^−6^) and ‘regulation of cell migration’ (P=0.00028).

In addition, miR-429, miR-663, miR-486-5p and miR-486-3p were verified in RNA samples derived from 231-P and 231-B cells by real-time PCR assay. Results showed that these miRNAs indeed had distinct expression patterns, indicating that they probably play important roles in bone metastasis of breast cancer cells (Fig. 3B).

### miR-429 suppresses invasion of 231-B cells in vitro

Previous reports showed that miR-429 could inhibit migration and metastasis of several types of cancers. Overexpression of miR-429 inhibits invasion and promotes apoptosis in esophageal carcinoma cells by targeting Bcl-2 and SP1 (19). miR-429 inhibits cells invasion by targeting Onecut2 in colorectal carcinoma (20). Our results were in line with the above investigations.

**Figure 4:**
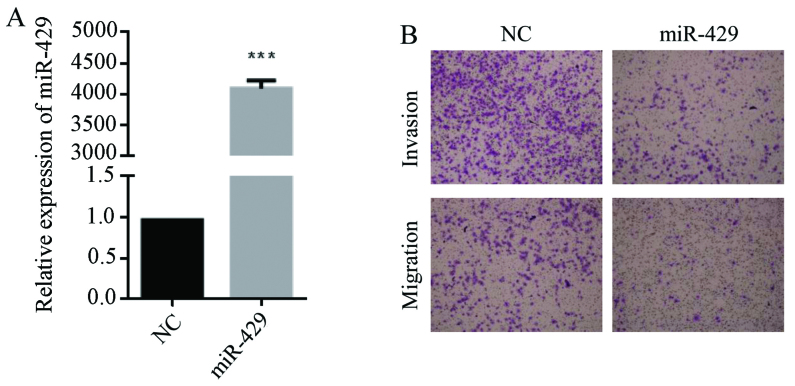
miR-429 inhibits migration and invasion of MDA-MB-231 cells *in vitro*. (A) Overexpression of miR-429 in 231-B cells, showing the efficiency of transfection. (B) Increased miR-429 significantly hampers the motility and invasiveness of 231-B cells *in vitro*.

We noted that miR-429 expression was significantly lower in 231-B cells than that of 231-P cells (Fig. 3B). We transfected miR-429 mimics into 231-B cells, which was verified by real-time PCR assay (Fig. 4A), and found that overexpression of miR-429 could dramatically reduce the migration and invasiveness of 231-B cells (Fig. 4B). Taken together, miR-429 probably regulated bone metastasis of breast cancer cells in a negative manner.

### miR-429 mediates inhibitory function by targeting multiple genes

**Figure 5:**
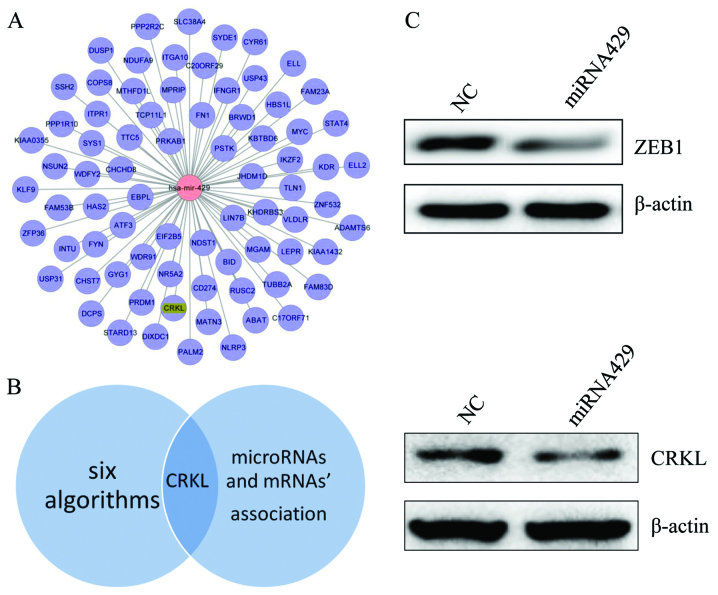
Search for the functional targets of miR-429. (A) The microRNAs and their potential targets detected in a global expression assay. (B) The map illustrates that CRKL is probably a new genuine target of miR-429 in bone metastasis of breast cancer cells through cross-evaluating results from *in silico* search and expression array. (C) Immunoblot assay shows that overexpression of miR-429 in 231-B cells results in reduced expression of ZEB1 and CRKL.

To gain insight into the roles of miR-429 inhibiting bone metastasis of MDA-MB-231 cells, several computational algorithms (including TargetScan, DIANA-microT, microRNA.org, miRDB, RNA22-HSA and PITA) were used to predict the candidate targets of miR-429, ZEB1, ZEB2, WIPF1, MARCKS, TRIM33, WASF3, CRKL, WAPAL and NTRK2 were predicted as candidate targets of miR-429 (Table I). Fig. 5A shows the possible regulation network between miRNAs and mRNAs. Then, we combined the *in silico* analysis of miR-429 targets and global transcriptional profile (Fig. 5B). Among all these results, CRKL was the target gene on both sides, indicating this gene was probably a fundamental node in controlling bone metastasis of breast cancer. Moreover, immunoblot assay showed that miR-429 reduced ZEB1 and CRKL significantly, which is a classical EMT inducer in breast cancer (Fig. 5C). Collectively, miR-429 negatively modulated several key invasion and metastasis inducers to suppress motility of breast cancer cells.

## Discussion

Breast cancer morbidity ranks the first accounting for 29% in all the women suffering cancer, and the mortality ranks the second accounting for 14% among the women cancer victims in western countries (21,22). Although great progress has been made toward preventing and treating breast cancer with surgical operation, chemotherapy, and radiation therapy, metastasis of breast cancer still remains one grave clinical challenge due to the limits of early detection and effective treatment. Breast cancer is likely to form distant metastases in lung, bone and liver (23). A myriad of factors influence malignant mammary cells to colonize in bones (24,25). In this investigation, we set up an *in vivo* model to study how microRNAs regulate bone metastases of breast cancer cells.

We established a bone metastasis model using MDA-MB-231 cells, and further miRNA array analysis revealed the altered expression of more than 100 miRNAs between 231-P and 231-B cells. Among these miRNAs, several have already been found to get involved in invasion and metastasis of tumors. miR-663 could inhibit proliferation and invasion of glioblastoma *in vitro* and *in vivo* by directly targeting PIK3CD, thus, reducing the expression of three downstream effectors: CCND1, MMP2 and MMP7 (26). On the other hand, in castration-resistant prostate cancer cells, miR-663 acted as OncomiR, which could promote invasion of malignant cells. Moreover, this miRNA correlated with TNM stage and could be used as an independent prognostic marker in clinical recurrence (27). Besides, miR-486-5p, expression of which in 231-B cells was decreased by 70% compared with that of 231-P cells, was recently reported to inhibit metastasis of lung cancer by reducing the protein level of ARHGAP5 (28). Taken together, most array results from comparison between 231-P and 231-B cells were in line with previous literature and these differentially expressed miRNAs are probably implicated in bone metastasis of breast cancer.

miR-429 is a member of the miR-200 family, four members of this family have been found to play important roles in the regulation of EMT in various types of tumors (29). EMT is widely accepted as an essential mechanism to prompt malignant cells to invade and colonize distant organs (30). In the present study, we showed that downregulation of miR-429 could promote bone metastasis of breast cancer, which is in line with previously reported results in several types of cancers. Besides in breast cancer, overexpression of miR-429 suppressed invasion and metastasis of colorectal carcinoma and ovarian cancer cells, respectively (20,31).

miRNAs usually function through base-pairing specific sequences located in 3′-UTRs of targeted genes to hinder translation of mRNA or enhance degradation of mRNA (32). ZEB1 is the most well studied target of miR-429, since this transcription factor is an important EMT inducer (33). Besides ZEB1, several candidate targets of miR-429 were also identified essential for invasion and metastasis in cancer models. For example, Onecut2 was proved to be one *bona fide* target of miR-429 in colon cancer (20). Combined analysis of expression array results and *in silico* target search revealed CRKL as a common candidate. CRKL protein is a kinase harboring SH2 and SH3 domains. CRKL transformed fibroblasts and received signals from BCR-ABL tyrosine kinase (34–36). CRKL influences integrin mediated adhesion to fibronectin (37). For cellular motility, CRKL enhanced motion through forming a complex with Cbl and C3G to relay signals in hematopoietic Ba/F3 cells (38). In addition to promoting invasion of cells, CRKL also could suppress caspase-8 mediated apoptosis, which was also essential for distant metastasis (39). Another research group also found the involvement of CRKL in integrin-triggered cell migration, and they further identified that CRKL performed by acting as downstream effector of Src (40). Considering that CRKL is important in proliferation, migration and evading apoptosis, we speculate that CRKL is probably essential in bone metastasis of breast cancer, however this needs further investigation.

In conclusion, we established an *in vivo* bone metastasis model of breast cancer, and we obtained a wealth of information on how mammary malignant cells invade and finally form overt metastases in bones. Further research is urgently needed to clarify the complicated mechanisms underlying this fatal pathological condition to provide valuable prevention and treatment targets.

## Figures and Tables

**Table I:** The candidate targets of miR-429, which were verified to play a vital role in the development of certain cancer, were predicted in six algorithms.

	TargetScan	DIANA-microT	microRNA.org	miRDB	RNA22-HSA	PITA
ZEB1	+	+	+	+	+	+
ZEB2	+	+	+	+	+	
WIPF1	+	+	+	+		+
MARCKS	+	+	+	+		+
TRIM33	+	+	+	+		+
WASF3	+	+	+	+		+
CRKL	+	+	+	+		+
WAPAL	+	+	+	+		+
NTRK2	+		+	+	+	+
